# Associations among Antibiotic and Phage Resistance Phenotypes in Natural and Clinical *Escherichia coli* Isolates

**DOI:** 10.1128/mBio.01341-17

**Published:** 2017-10-31

**Authors:** Richard C. Allen, Katia R. Pfrunder-Cardozo, Dominik Meinel, Adrian Egli, Alex R. Hall

**Affiliations:** a Institute for Integrative Biology, Department of Environmental Systems Science, ETH Zürich, Zürich, Switzerland; b Division of Clinical Microbiology, University Hospital Basel, Basel, Switzerland; c Applied Microbiology Research, Department of Biomedicine, University of Basel, Basel, Switzerland; McMaster University

**Keywords:** *Escherichia coli*, antibiotic resistance, bacteriophages, evolution, microbial ecology, plasmids

## Abstract

The spread of antibiotic resistance is driving interest in new approaches to control bacterial pathogens. This includes applying multiple antibiotics strategically, using bacteriophages against antibiotic-resistant bacteria, and combining both types of antibacterial agents. All these approaches rely on or are impacted by associations among resistance phenotypes (where bacteria resistant to one antibacterial agent are also relatively susceptible or resistant to others). Experiments with laboratory strains have shown strong associations between some resistance phenotypes, but we lack a quantitative understanding of associations among antibiotic and phage resistance phenotypes in natural and clinical populations. To address this, we measured resistance to various antibiotics and bacteriophages for 94 natural and clinical *Escherichia coli* isolates. We found several positive associations between resistance phenotypes across isolates. Associations were on average stronger for antibacterial agents of the same type (antibiotic-antibiotic or phage-phage) than different types (antibiotic-phage). Plasmid profiles and genetic knockouts suggested that such associations can result from both colocalization of resistance genes and pleiotropic effects of individual resistance mechanisms, including one case of antibiotic-phage cross-resistance. Antibiotic resistance was predicted by core genome phylogeny and plasmid profile, but phage resistance was predicted only by core genome phylogeny. Finally, we used observed associations to predict genes involved in a previously uncharacterized phage resistance mechanism, which we verified using experimental evolution. Our data suggest that susceptibility to phages and antibiotics are evolving largely independently, and unlike in experiments with lab strains, negative associations between antibiotic resistance phenotypes in nature are rare. This is relevant for treatment scenarios where bacteria encounter multiple antibacterial agents.

## IMPORTANCE

Rising antibiotic resistance is making it harder to treat bacterial infections. Whether resistance to a given antibiotic spreads or declines is influenced by whether it is associated with altered susceptibility to other antibiotics or other stressors that bacteria encounter in nature, such as bacteriophages (viruses that infect bacteria). We used natural and clinical isolates of *Escherichia coli*, an abundant species and key pathogen, to characterize associations among resistance phenotypes to various antibiotics and bacteriophages. We found associations between some resistance phenotypes, and in contrast to past work with laboratory strains, they were exclusively positive. Analysis of bacterial genome sequences and horizontally transferred genetic elements (plasmids) helped to explain this, as well as our finding that there was no overall association between antibiotic resistance and bacteriophage resistance profiles across isolates. This improves our understanding of resistance evolution in nature, potentially informing new rational therapies that combine different antibacterials, including bacteriophages.

## INTRODUCTION

Antibiotics and bacteriophages are common antibacterial agents (stressors) in natural and clinical environments (1), selecting for diverse resistance mechanisms in bacteria (2, 3). However, it is not yet clear whether or how bacterial resistance to antibiotics and phages are associated. For example, if phage-resistant bacteria also have relatively high or low antibiotic resistance, then phage selection could drive changes in the distribution of antibiotic resistance phenotypes in natural and clinical populations (4). This is important for our basic understanding of how bacteria adapt to these common selection pressures, but also in the context of antibacterial treatments. Nonrandom associations among resistance phenotypes could compromise novel therapies exploiting phages alone or in combination with antibiotics (5), because prior exposure to one stressor could prime the bacterial population to resist others. In contrast, if such associations among resistance phenotypes are rare, this could pave the way for treatment strategies employing multiple stressors or types of stressors with independent resistance mechanisms.

Correlations among resistance phenotypes across bacterial genotypes have been demonstrated for some combinations of antibiotics, either because the same allele pleiotropically alters susceptibility to multiple antibiotics (3, 6, 7) or because multiple resistance alleles cooccur on the same genome or plasmid (8). Such associations permit one antibiotic to coselect for resistance to others, and this is implicated in the emergence and persistence of multiresistant pathogens (8, 9). Associations among resistance phenotypes can also be negative, which could potentially be exploited to design treatments that minimize the risk of resistance evolution (10, 11). Although *in vitro* experiments with resistant mutants isolated from lab strains suggest that such negative associations are quite common (12), it remains unclear whether these effects apply in natural populations (13, 14). In particular, exploiting these effects in novel multidrug treatments depends critically on whether they also apply to other types of resistance elements such as plasmids that are central to the evolution and spread of resistance in pathogens such as *Escherichia coli* (15). Antibiotic resistance can also be associated with resistance to other types of stressors, such as heavy metals (16). In the case of resistance to phages, recent *in vitro* studies revealed strong associations for specific antibiotic-phage pairs, due to pleiotropic effects of the same allele on resistance to both stressors (17) or physical association of genes affecting both types of resistance (18). Although these studies involved a limited number of antibiotic-phage combinations *in vitro*, both types of associations are also plausible outside the laboratory: infection by some phages involves bacterial proteins also involved in antibiotic resistance (19, 20), and populations exposed to antibiotics are frequently also parasitized by phages (19). Despite this, we lack a quantitative understanding of how resistance phenotypes against different stressors, and particularly different types of stressors, are associated in natural and clinical bacteria.

To address this, we studied a library of 94 phylogenetically diverse *Escherichia coli* isolates (21, 22) taken from healthy and sick humans and animals over a wide geographical area over several decades and carrying diverse plasmids (17, 22–24). For each isolate, we measured (i) antibiotic resistance as the 90% inhibitory concentration (IC_90_) in liquid culture for 10 antibiotics from diverse classes (see Table S1 in the supplemental material) and (ii) phage resistance as the ratio of bacterial growth in the presence to that in the absence of each of 14 phage species (Table S2). We used phages from seven families, mostly from the prevalent *Caudovirales* order, the principal order considered for phage therapy (5). We used genome assemblies to assess the influence of nucleotide similarity in conserved regions of the genome (core genome multilocus sequence typing [cgMLST]-based phylogeny) and similarity of plasmid replicon profiles among isolates on their resistance phenotypes. We assessed the strength of nonrandom associations between resistance phenotypes while accounting for core genome phylogenetic relatedness (25, 26). Our primary aim here was to test for associations among resistance phenotypes across different antibacterial agents, rather than investigating the mechanisms behind individual associations. Nevertheless, our data set allowed us to make predictions about the genetic basis of some of the observed associations, and we used genetic knockouts and experimental evolution to investigate these in more detail.

10.1128/mBio.01341-17.4 TABLE S1  Phages used in this study. Download TABLE S1, DOCX file, 0.1 MB. Copyright © 2017 Allen et al. 2017 Allen et al. This content is distributed under the terms of the Creative Commons Attribution 4.0 International license.

**FIG 1:**
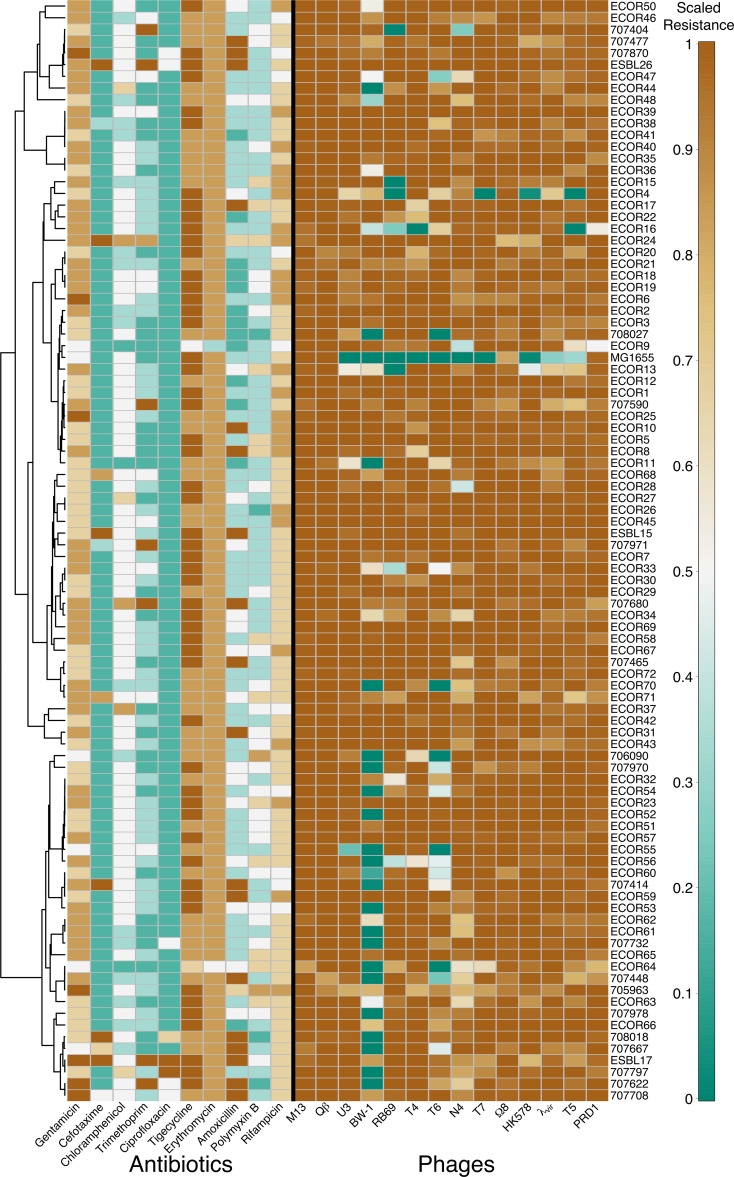
Resistance profiles of all isolates grouped by their phylogenetic relationships. In the heat map, brown cells are relatively resistant and green cells are susceptible for both antibiotics (left) and phages (right). Antibiotic resistance is scaled so that resistance is 0.5 when IC_90_ equals the breakpoint (Table S1). Phage resistance shows the ratio of growth in the presence to that in the absence of phages, truncated above one and below zero. Scaled values are used only for this representation. The dendrogram (left) is derived from the phylogeny in Fig. S1a.

10.1128/mBio.01341-17.5 TABLE S2  Antibiotics used in this study. Most breakpoint values correspond to clinical breakpoints taken from the EUCAST website (http://www.eucast.org), v7.1. Where two values of the breakpoint are given, susceptibility is defined by EUCAST as an MIC of ≤the lower value and resistance by an MIC of >the higher value; we used the value in bold to scale resistances in Fig. 1. (a) These antibiotics have high levels of resistance in *E. coli* and have no published EUCAST breakpoint, so breakpoints were taken as the midpoints of the MIC distribution from past work (I. Stock and B. Wiedemann, Diagn Microbiol Infect Dis 33:187–199, 1999). DHFR, dihydrofolate reductase. Download TABLE S2, DOCX file, 0.05 MB. Copyright © 2017 Allen et al. 2017 Allen et al. This content is distributed under the terms of the Creative Commons Attribution 4.0 International license.

## RESULTS

### Phylogenetic distribution of resistance phenotypes.

Bacterial isolates varied in their resistance to antibiotics and phages (Fig. 1), although for phages most isolate-phage interactions did not result in inhibition of bacterial growth. This is consistent with the idea that phages are usually specific for particular host genotypes or strains. For both phage resistance and antibiotic resistance, more closely related isolates tended to have more similar resistance profiles (Euclidean distance between resistance scores across antibiotics or phages; Mantel test for correlation with patristic genetic distances: antibiotic resistance correlation = 0.05, *P* < 0.05; phage resistance correlation = 0.11, *P* < 0.01). Despite this, it was not the case that phylogenetically close isolates had similar overall resistance measured as average resistance across all antibiotics (*P* [Mantel] > 0.1; *P* [Pagel’s λ] > 0.5) or phages (*P* [Mantel] > 0.1; *P* [Pagel’s λ] > 0.5), suggesting that our isolates do not fall into generally more or less resistant clades for either type of stressor. For individual stressors, associations with phylogenetic distance were rare after accounting for multiple testing (Materials and Methods). This was also the case when using an alternative (Pasteur) MLST scheme (10) (see Fig. S1 in the supplemental material).

10.1128/mBio.01341-17.2 FIG S1  Phylogenetic trees of the isolate library used in this paper. (a) Nucleotide tree constructed using the concatenated sequences of 1,424 core genome (cg) loci (1,110,238 bp) for which sequences were available for the whole library. The tree is rooted according to the root position in the Pasteur MLST tree. (b) Nucleotide tree constructed using the concatenated sequences of seven loci of the Pasteur MLST scheme (3,495 bp; *uidA* not used due to missing allele sequences). The tree is rooted according to the position of the *Escherichia fergusonii* outgroup. In both cases, the trees were constructed using RAxML. Node labels show bootstrap support; 100% bootstrap support labels are omitted, and bootstrap values below 85% are highlighted in red. Isolates are grouped into the ECOR groups according to the analysis of Sahl et al. (J. W. Sahl, M. N. Matalka, and D. A. Rasko, Appl Environ Microbiol 78:4884–4892, 2012, https://doi.org/10.1128/AEM.00929-12), which uses an MLST scheme that differs from both the cgMLST scheme and the Pasteur MLST scheme. The two trees show a high degree of similarity based on a Mantel test of pairwise genetic distances (*r* = 0.84, *P* < 0.05) and a Robinson-Foulds distance of 140. Our analysis of resistance phenotypes, reported in the text using the cgMLST tree to measure phylogenetic relatedness, is also similar when we instead use the Pasteur MLST tree: overall phage (*r* = 0.088, *P* < 0.05) and antibiotic (*r* = 0.080, *P* < 0.05) resistances are more similar between closely related isolates. As for the cgMLST tree, resistance to phage BW-1 showed a significant phylogenetic distribution on the Pasteur MLST tree (λ = 0.35, *P* < 0.001), but additionally so did trimethoprim resistance (λ = 0.45, *P* < 0.05). Download FIG S1, TIF file, 2.3 MB. Copyright © 2017 Allen et al. 2017 Allen et al. This content is distributed under the terms of the Creative Commons Attribution 4.0 International license.

### Nonrandom associations between resistance phenotypes.

**FIG 2:**
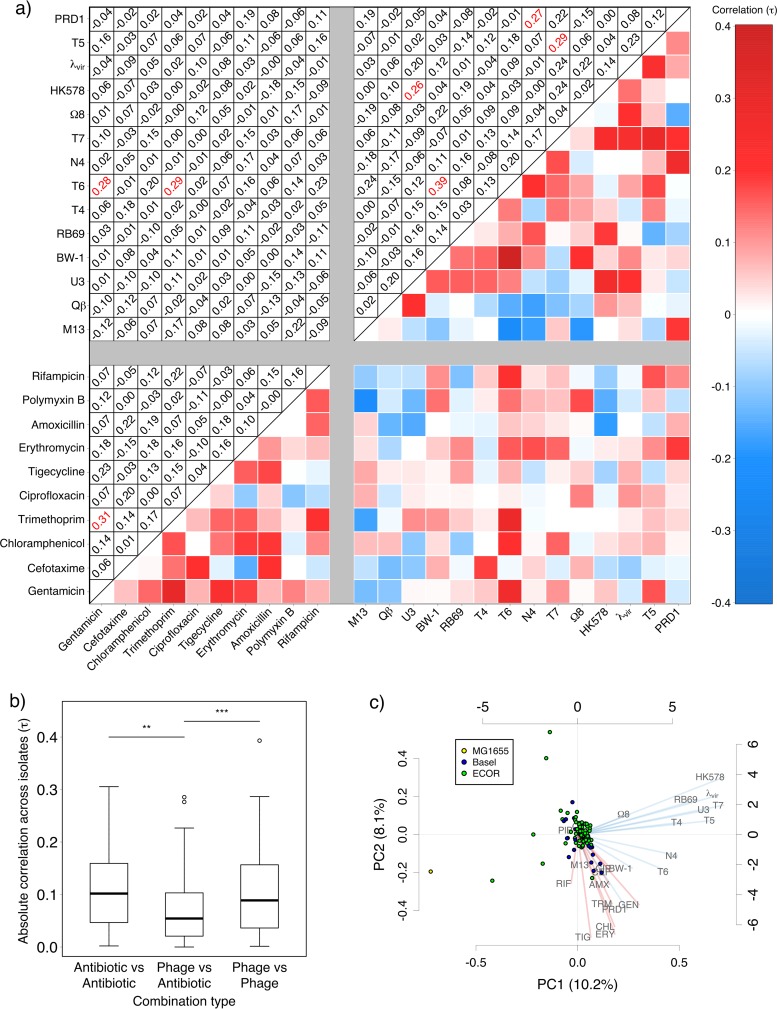
Correlations between resistance phenotypes. (a) Correlations between resistances to each pair of stressors (analyzed as phylogenetically independent contrasts); shading in the lower triangle gives correlation strength and sign; correlation coefficients are given in the upper triangle, with significant values (α = 0.05 after adjusting for multiple testing) in red. (b) Magnitude of correlation coefficients for stressor pairs within and between stressor types. (c) Principal-component analysis, showing isolates as points on the first two components (colored by origin: isolates from the ECOR collection, clinical isolates from the University Hospital Basel, or laboratory strain K-12 MG1655) and showing loadings for phages (blue) and antibiotics (red) as lines radiating from the center.

Among individual pairs of resistance phenotypes, we found several significant associations (after adjusting for multiple testing [Fig. 2a]), including antibiotic-antibiotic, phage-phage, and phage-antibiotic pairs. Significant associations were all positive, providing no evidence that resistance to one stressor is linked to susceptibility to others. These pairwise associations were on average stronger for pairs of stressors of the same type (phage-phage, χ^2^_1_ = 11.23, *P* < 0.001; antibiotic-antibiotic, χ^2^_1_ = 10.36, *P* < 0.01) than for phage-antibiotic combinations (Fig. 2b). Consistent with this, there was also no association between overall phage resistance and antibiotic resistance profiles across isolates (partial Mantel correlation = 0.002, *P* > 0.5). This indicates that, despite rare associations among some individual pairs of phage and antibiotic resistance phenotypes, susceptibility to antibiotics and that to phages evolve independently from each other overall. Principal-component analysis (PCA) also showed that resistance to the two types of stressors was approximately orthogonal across isolates, as demonstrated by the grouping of phage (blue) and antibiotic (red) resistance variation for the first two components (Fig. 2c). The PCA also reveals some interesting exceptions, such as the plasmid-dependent phage PRD1, for which variation of resistance was more similar to antibiotic resistance phenotypes than to other phage resistance phenotypes.

When only very few isolates are susceptible to a given phage, as is the case for many phages in our data set, associations with susceptibility to other stressors can potentially be driven by only a small number of susceptible isolates. While these associations are still biologically meaningful, it is relevant to distinguish them from associations driven by variable susceptibility across many isolates. We tested this by identifying cases where only a few (≤5) isolates were susceptible and testing the effect of excluding individual isolates (Text S1). In all cases, this had only small effects on the strength of the association (Kendall’s tau [Table S3]), although in some cases associations were no longer significant after accounting for multiple testing. This indicates that some phage-phage associations in our data set were strongly influenced by a relatively small number of isolates being susceptible to both phages.

10.1128/mBio.01341-17.1 TEXT S1  Supplemental materials and methods. Download TEXT S1, DOCX file, 0.04 MB. Copyright © 2017 Allen et al. 2017 Allen et al. This content is distributed under the terms of the Creative Commons Attribution 4.0 International license.

10.1128/mBio.01341-17.6 TABLE S3  Testing the influence of individual phage-sensitive isolates on observed associations. Effects of removing individual phage-sensitive (resistance < 0.6) isolates are given for correlations where five or fewer isolates were sensitive to one or both of the stressors involved. Download TABLE S3, DOCX file, 0.1 MB. Copyright © 2017 Allen et al. 2017 Allen et al. This content is distributed under the terms of the Creative Commons Attribution 4.0 International license.

### Plasmid profiles predict antibiotic resistance but not phage resistance phenotypes.

**FIG 3:**
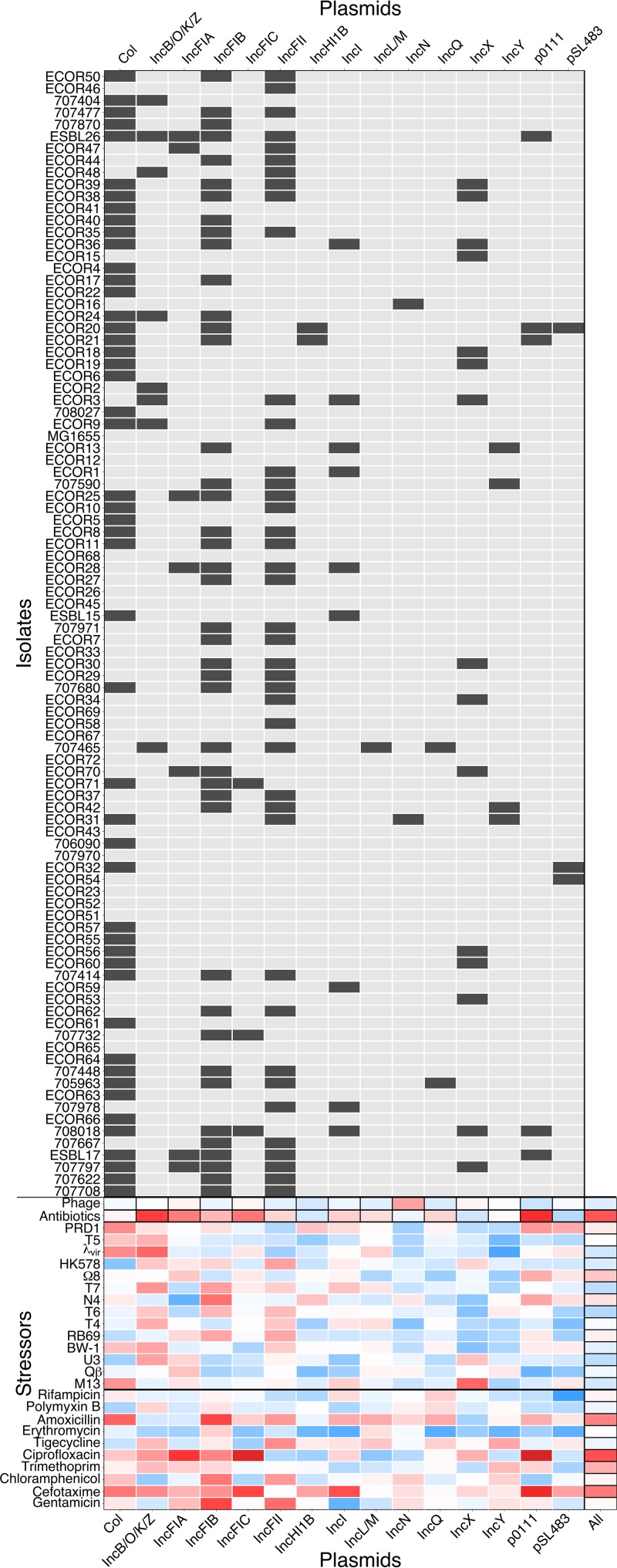
Plasmid profiles and association with resistance phenotypes. (Top) Presence (dark gray) of each plasmid replicon in each isolate. (Bottom) Correlations between individual resistance phenotypes and plasmid replicons (Kendall correlation across all isolates). Correlations for aggregate measures of resistance and/or plasmid profile (indicated with black-outlined boxes) were tested by Mantel tests of pairwise distances among isolates. Red indicates positive correlation, and blue indicates negative correlation, with depth of color indicating strength.

Plasmids are important determinants of resistance against antibiotics (8) and some phages (27). We therefore asked whether the plasmid content of our isolates could explain their multivariate resistance phenotypes. We found that isolates with similar plasmid replicon profiles (Fig. 3) also had similar antibiotic resistance profiles (partial Mantel correlation = 0.22, *P* < 0.001). In contrast, similarity of phage resistance profiles across isolates showed no association with similarity of their plasmid profiles (partial Mantel correlation = −0.040, *P* > 0.5). This is surprising given that some phages (M13 and Qβ) require F-plasmid-encoded proteins for infection and that several of our isolates carry F plasmids. However, these phages did not cause significant reduction in growth for any of the isolates tested here, even though we cultured them and determined their titers successfully using an F^+^ variant of K-12 MG1655. Lack of growth inhibition in these combinations was also evident by plaque assays on agar (Text S1). This suggests that even for phages known to be plasmid dependent on permissive hosts, there are other constraints on infection that precluded an association with plasmid content across our isolates. Crucially, plasmid profile here is not simply a proxy for phylogenetic relatedness: phylogenetic distances were not correlated with differences in plasmid profile (correlation = −0.0084, *P* > 0.1) or carriage of individual replicon types. These results were qualitatively unchanged when we excluded nonconjugative plasmids (Col, IncQ, and p0111 [28]) from the analysis: antibiotic resistance (partial Mantel correlation = 0.18, *P* < 0.05), but not phage resistance (partial Mantel correlation = −0.031, *P* > 0.5) or phylogenetic distance (correlation = −0.024, *P* > 0.1), was still correlated with plasmid profile.

Among individual plasmid replicon types, only some were correlated with antibiotic resistance phenotypes across our bacterial isolates (Fig. 3; Tables S4 and S5). We found strong associations between possession of plasmid p0111, originally isolated from an enterohemorrhagic *E. coli* strain (29), and resistance to both ciprofloxacin (tau = 0.50, *P* < 0.001) and cefotaxime (tau = 0.36, *P* < 0.001); this may contribute to the weak positive correlation between resistances to these antibiotics across isolates (Fig. 2a). Ciprofloxacin resistance was also associated with possession of IncFIA (tau = 0.27, *P* < 0.05) and IncFIC (tau = 0.46, *P* < 0.001), consistent with past observations of quinolone resistance and extended-spectrum β-lactamases being associated with F plasmids (30, 31). Because the same resistance gene is not always associated with the same plasmid or replicon, some correlations across isolates can be explained by shared resistance genes but not shared plasmid profiles. This is the case for the correlation between gentamicin resistance and trimethoprim resistance across isolates (Fig. 2a): isolates ESBL17 and 707622 show the highest resistance to both antibiotics and both have closely related genes for resistance against them, but these genes appear to be colocalized to different plasmids in the two isolates (Table S6).

10.1128/mBio.01341-17.7 TABLE S4  Correlation of individual antibiotic resistance phenotypes with aggregated plasmid profile. Measured with partial Mantel tests (1,000 permutations) corrected with genetic distance from the cgMLST tree. The contribution of individual plasmid types to cefotaxime, ciprofloxacin, and amoxicillin resistance is shown in Table S5. The same qualitative results are obtained if the Pasteur tree is used to generate genetic distances. Download TABLE S4, DOCX file, 0.05 MB. Copyright © 2017 Allen et al. 2017 Allen et al. This content is distributed under the terms of the Creative Commons Attribution 4.0 International license.

10.1128/mBio.01341-17.8 TABLE S5  Association with individual plasmid replicons for the antibiotic resistance phenotypes correlated with overall plasmid profile. Nonparametric (Kendall) correlation tests were performed on the phylogenetically independent contrasts of the resistance trait of interest and the plasmid presence or absence data (coded as 0 and 1 for presence or absence, respectively, and thus producing noninteger values for the PICs). Phylogenetically independent contrasts were produced using the cgMLST scheme tree. Multiple testing correction is performed within the treatment groups (i.e., using *n* = 10). If the Pasteur MLST tree is used to generate genetic distances, cefotaxime resistance is significantly positively correlated with IncFIC plasmids; otherwise, the same associations are seen. Download TABLE S5, DOCX file, 0.1 MB. Copyright © 2017 Allen et al. 2017 Allen et al. This content is distributed under the terms of the Creative Commons Attribution 4.0 International license.

10.1128/mBio.01341-17.9 TABLE S6  Plasmids matching the resistance regions for trimethoprim and gentamicin in isolates ESBL17 and 707622. Each row gives information for one plasmid sequence found to have high identity with the trimethoprim- or gentamicin-resistance-determining regions in either isolate (determined using ResFinder; see Text S1). Shaded rows highlight two plasmids (CP010149 and LN850163) with high identity to both gentamicin- and trimethoprim-resistance-determining regions in both isolates. Despite this, the two plasmid sequences with high identity to both isolates did not have replicon profiles that fully matched the replicon profile of both isolates. This suggests that these resistance genes are associated with different plasmids or with different replicons. Download TABLE S6, DOCX file, 0.1 MB. Copyright © 2017 Allen et al. 2017 Allen et al. This content is distributed under the terms of the Creative Commons Attribution 4.0 International license.

The importance of plasmid-borne resistance can also help to explain the finding that, across all pairs of stressors, we detected no significant negative associations across isolates. Our experiment included several antibiotics previously associated *in vitro* with resistance mechanisms conferring collateral sensitivity to other antibiotics, such as gentamicin resistance alleles causing increased sensitivity to polymyxin B and chloramphenicol (7). The lack of negative correlations here can be explained by these natural and clinical isolates having variable resistance according to their carriage of resistance mechanisms different from those arising by chromosomal mutation *in vitro*, such as plasmids carrying multiple resistance genes (e.g., Table S6).

### Pleiotropic effects of modifying phage receptors.

Some of the nonrandom associations involving phages (Fig. 2a) can be interpreted in light of information about resistance mechanisms or phage life cycles. For example, susceptibility to phages BW-1 and T6 was positively correlated across isolates. These phages are both T4-like viruses and therefore have similar life cycles and morphologies (32). Susceptibility to phage T6 was also correlated with susceptibility to trimethoprim and gentamicin. We found that deleting the *tsx* gene encoding the nucleotide channel used as a receptor by T6 (33) conferred resistance to T6 as expected and also increased resistance to both gentamicin and phage BW-1 but not trimethoprim (Fig. S2). This demonstrates that phage receptor modification can pleiotropically alter other resistance phenotypes, including those conferring resistance to other types of stressors. In other stressor combinations, we found only weak associations across isolates, despite cross-resistance being known to occur for resistant mutants isolated *in vitro*, such as phages T4 and T7 (34). As for antibiotic resistance, this suggests that correlated resistance phenotypes observed for mutants isolated from a single strain *in vitro* do not necessarily translate to natural or clinical populations.

10.1128/mBio.01341-17.3 FIG S2  Changes in antibiotic and phage resistance associated with deletion of *tsx* gene. (a) Antibiotic resistance of the Δ*tsx* knockout and the ancestral strain measured as described in the text (IC_90_ values reported in units of ranked concentrations; ranks 5 and 6 for trimethoprim correspond to IC_90_s of 0.5 μg/ml and 1 μg/ml, respectively; ranks 6 and 7 for gentamicin correspond to IC_90_s of 4 μg/ml and 8 μg/ml, respectively; rank 8 is an IC_90_ above 8 μg/ml; *n* ≥ 5 in all cases). (b) Phage resistance measured as described in the text (*n* ≥ 10 in all cases). Download FIG S2, TIF file, 2 MB. Copyright © 2017 Allen et al. 2017 Allen et al. This content is distributed under the terms of the Creative Commons Attribution 4.0 International license.

### Predicting genes involved in resistance.

**FIG 4:**
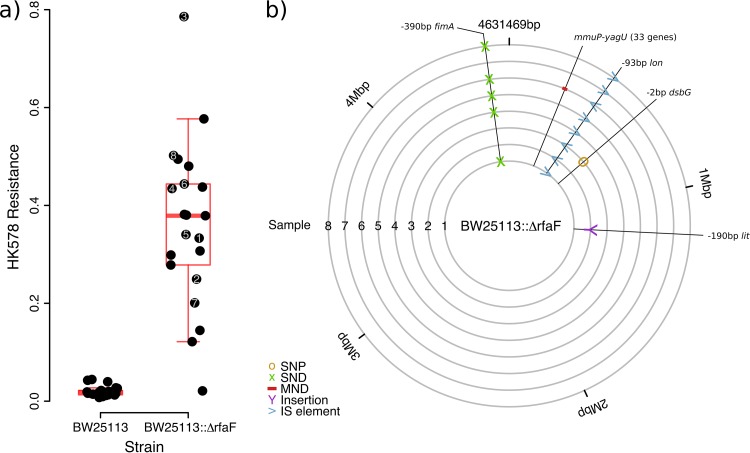
Deleting *rfaF* potentiates resistance evolution against HK578 via mutations near *lon*. (a) Resistance measured as the ratio of population growth in the presence to that in the absence of HK578 for each of ≥21 replicates inoculated from independent overnight cultures. (b) Genetic changes from the Δ*rfaF* ancestor of eight HK578-resistant genotypes isolated from the end of the experiment (marked in panel a). Mutants are marked with the genes affected; some mutations are intergenic and are marked with the closest gene downstream, as well as their position relative to the start of the open reading frame. SNP, single nucleotide polymorphism; SND, single nucleotide deletion; MND, multinucleotide deletion; IS, insertion sequence.

For some phages, mechanisms of infection and resistance are unknown. We therefore asked whether we could identify bacterial genes involved in these processes by exploiting information about associations with resistance to other phages for which more information is available. Specifically, the receptor and resistance mechanisms for phage HK578 are unknown, but we found positive correlations with resistance to U3, λ, T7, N4, and RB69 (correcting for phylogeny with either Pasteur or core genome MLST profiles). From information about these phages, we selected 9 candidate genes (Text S1) that could be involved in HK578 infection or resistance. Of these genes, we found that deleting *rfaF* (also known as *waaF*) altered population-level sensitivity to HK578. *rfaF* is involved in lipopolysaccharide biosynthesis and influences infection by both T7 and U3 (35, 36). However, individual populations of this knockout varied considerably in their responses to this phage, potentially indicating the stochastic appearance of additional phage resistance mutations at different rates across replicate populations (37). We therefore carried out a second experiment with greater replication and tested for changes in HK578 sensitivity of the Δ*rfaF* knockout during the experiment (Fig. 4a). Colonies isolated from 19 out of 20 Δ*rfaF* populations that had been exposed to HK578 were HK578 resistant in a streak test (38), whereas both the Δ*rfaF* ancestor and colonies from Δ*rfaF* populations grown in the absence of HK578 were susceptible. Populations with intact *rfaF* that were exposed to HK578 did not survive the experiment (Fig. 4a). This indicates that *rfaF* knockout potentiates the evolution of resistance to this phage, rather than conferring it directly. Whole-genome sequencing of clones (Fig. 4b) from this experiment revealed parallel insertions of an IS*186* insertion sequence in the regulatory region of *lon* (*capR*). *lon* is involved in regulation of the capsule, and mutations here can produce mucoidy and resistance to λ phage (39). Consistent with this, every resistant mutant in this experiment was mucoid, whereas the Δ*rfaF* ancestor was not, even though *rfaF* mutation can cause mucoidy in certain backgrounds and under certain conditions (40). Thus, associations among resistance phenotypes can generate predictions about genes involved in uncharacterized resistance mechanisms.

## DISCUSSION

Associations among resistance phenotypes are known to be central to the success of some promising novel approaches to treatment, such as combining antibiotics in ways that minimize the spread of resistance (41) and applying phages as a complement or alternative to antibiotics (5). While previous work with mutants isolated *in vitro* from lab strains showed various types of associations, including negative associations (6, 7, 42), our results suggest that any nonrandom associations among pairs of resistance phenotypes across natural and clinical isolates tend to be positive. We found no significant negative associations among resistance phenotypes, even for antibiotic combinations that exhibit collateral sensitivity across mutants isolated *in vitro* (7, 27). This discrepancy is likely due to resistance in nature being frequently linked to different types of resistance genes (compared to those responsible for *de novo* resistance evolution in lab strains), in particular those carried on multiresistance elements such as plasmids (16, 43). Consistent with this, some antibiotic resistance phenotypes in our data set were linked to possession of particular plasmid replicons. We found that associations among resistance phenotypes could also result from pleiotropic effects of individual resistance alleles including, surprisingly, an effect of mutating the phage T6 receptor on resistance to gentamicin. Thus, our data suggest that nonrandom associations between resistance phenotypes can occur by multiple mechanisms and across stressor types, and this may modify pathogen responses to treatment in important ways. Additionally, we found that associations among resistance phenotypes can be used to predict genes involved in uncharacterized resistance mechanisms, which we demonstrated directly by identifying genes involved in resistance to phage HK578.

Despite our finding that nonrandom associations between resistance phenotypes exist for some combinations of stressors, across the entire data set such associations were rare. This is consistent with the high diversity and specificity of potential mechanisms of resistance against antibiotics and phages (2, 3). Nevertheless, we observed an overall trend that resistance phenotypes were on average more closely correlated across isolates within stressor types (antibiotic-antibiotic or phage-phage combinations) than across stressor types (phage-antibiotic). This indicates that the interesting associations observed previously for some specific antibiotic-phage pairs (27, 42) do not reflect a general association between the two types of resistance. The independent evolution of antibiotic and phage resistance profiles can be explained at least partially by our finding that antibiotic resistance, but not phage resistance, was strongly linked to plasmid profiles. Given that several of the key types of mechanisms of resistance to antibiotics and phages are conserved across bacterial species (e.g., target modification, resistance plasmids, and receptor modification) (2, 3), we predict that this independent evolution of the two types of resistance phenotypes may also apply in other bacterial species.

Our finding that phage and antibiotic resistance phenotypes are largely evolving independently suggests that phages naturally present in clinical or environmental settings are unlikely to drive the spread of antibiotic resistance and similarly that antibiotic use in medicine or agriculture is unlikely to drive changes in phage resistance. This is promising for the development of phage therapy and treatments based on phage-antibiotic combinations (5), as they are unlikely to suffer from cross-resistance between the two components, more often seen between antibiotics (6–8). We note that variable phage resistance in our library can result from bacteria having adapted to evade phage infection or because phages have evolved to specialize on some hosts rather than others (“non-host resistance” [44]). Any future work aimed at testing for cross-resistance resulting from bacterial adaptation to particular phage therapy treatments should focus on the former. More generally, the different types of associations observed across pairs of antibacterial agents in studies with lab-isolated mutants and in the natural and clinical isolates in our study suggest that the success of promising strategies like collateral sensitivity and phage-antibiotic treatments will depend critically on which resistance mechanisms (genes or plasmids) are circulating in the local pathogen population. This type of information is increasingly available through genomic/non-culture-based monitoring methods (e.g., 45 and 46).

## MATERIALS AND METHODS

### Organisms and growth conditions.

Our library of isolates included 70 isolates from the *E. coli* Reference (ECOR) Collection (17, 22, 47); ECOR isolates 14 and 49 were excluded based on similarity of phylogeny, metadata, and plasmid profile to other isolates in this collection. We supplemented this with 23 clinical isolates from the University Hospital, Basel, Switzerland, all isolated from urine samples of patients with urinary tract infections in 2016. All clinical samples were anonymized, and no clinical information was provided with the samples. We also included K-12 MG1655, giving 94 isolates in total. For investigation of genes involved in HK578, T6, and BW-1 infection and gentamicin or trimethoprim resistance, we used the Keio collection of single-gene knockouts of *E. coli* BW25113 (48). We stored isolates in 25% glycerol at −80°C. For routine culturing, we used lysogeny broth (LB) supplemented with 2 mM CaCl_2_ and 2 mM MgSO_4_. We used 1/10-diluted LB for overnight culturing prior to resistance assays to attain an appropriate number of cells per inoculated culture. Prior to phage resistance assays, we amplified and determined the titers of all phages (see Table S1 in the supplemental material) using K-12 MG1655 as a host (unless otherwise specified) and using the overlay method on LB agar plates. We then stored phages at 4°C during the experiments and at −80°C with 10% glycerol for longer-term storage.

### Measuring susceptibility of isolates to phages and antibiotics.

We estimated antibiotic resistance from bacterial growth observed across a 2-fold broth dilution series of each antibiotic (49), calculating the 90% inhibitory concentration (IC_90_) as the lowest concentration above which growth did not exceed 10% of that observed in the absence of antibiotics. Concentrations were ranked 1 to 5 to give the same linearized scale across antibiotics; if no concentration inhibited growth by 90% compared with the control, we assigned a rank of 6. We estimated phage resistance as the ratio between the density of cultures with the phage (2 × 10^6^ PFU per ml) and the median of the control cultures for that isolate (grown in the absence of phages or antibiotics) (50, 51). Further details are given in Text S1.

### Genome sequences of natural and clinical isolates.

We downloaded assemblies for the ECOR collection from EnteroBase (http://enterobase.warwick.ac.uk/species/index/ecoli). Assemblies were predominantly those from the Sanger Centre, or the FDA (Food and Drug Administration) when Sanger Centre assemblies were unavailable or lacked coverage for key regions. For the Basel isolates and for ECOR 72 (for which no assembly was available), we carried out whole-genome sequencing at the University Hospital Basel (Text S1). We used the pipeline at EnteroBase to generate a core genome MLST (cgMLST) profile for all isolates (52). We concatenated alleles for the 1,424 loci with nucleotide sequences reported for each isolate and used this alignment to generate a rooted tree using RAxML (53). Full details, including the alternate Pasteur MLST scheme, are given in Text S1.

### Plasmid replicon profiles.

We used PlasmidFinder (13, 14) on the genome assemblies for each isolate (with an identity threshold of 75%). We then grouped plasmids at the level of incompatibility groups (or subgroups for F plasmids). We also included plasmids similar to p0111 and pSL483 which were found multiple times but are not typed. Plasmid profiles were made up of presence/absence data for 15 plasmid types (Text S1).

### **Sequencing phage-resistant mutants of** *E. coli* BW25113.

For preliminary experiments, we chose 9 candidate genes (*galU*, *lamB*, *nfrA*, *ompF*, *fhuA*, *rfaP*, *rfaG*, *rfaF*, and *gmhA*) that are known to be involved in phenotypes for resistance to other phages that were correlated with resistance to HK578 in the analysis with either the cgMLST or the Pasteur MLST phylogeny. We tested these for inhibition by phage HK578 in liquid culture and then proceeded with a larger experiment with only the *rfaF* knockout (Fig. 4a). Of the 19 populations that we found to have acquired resistance to HK578, we randomly selected 8 populations, isolating a single colony from each. We then sequenced these clones along with the ancestral BW25113::Δ*rfaF* at the Genomic Diversity Centre, ETH Zürich (Text S1). We used breseq (19, 20) to analyze the sequencing data, mapping paired reads to the annotated BW25113 reference genome (Text S1). Unassigned junction evidence was manually resolved by examining the read evidence as recommended in breseq documentation (19).

### Statistics and analysis.

Unless otherwise stated, we performed all analysis in R (54). We imported the phylogenetic tree into R using phytools (55). To test the strength of the associations between phage resistance profiles and phylogenetic similarity and between antibiotic resistance profiles and phylogenetic similarity, we first computed the pairwise Euclidean distances between isolates in terms of resistance across all phages or antibiotics. We then used Mantel tests (ncf package [56]) to determine the association of these distances with (patristic) phylogenetic distances among the same pairs of isolates. We also tested whether overall resistance (averaged across all phages or antibiotics for each isolate) was more similar for more closely related isolates using both Mantel tests and by calculating Pagel’s λ using fitContinuous or fitDiscrete (with equal rates) functions in the GEIGER package (57). For individual resistance phenotypes, we estimated phylogenetic signal by calculating Pagel’s λ and adjusting *P* values to account for multiple testing using the Holm-Bonferroni method (sequential Bonferroni). We found several associations with *P* < 0.05 here, but after accounting for multiple testing, only phage BW-1 showed significant association with phylogenetic distance (λ = 0.27, adjusted *P* < 0.001). We used the same sequential Bonferroni correction elsewhere whenever multiple testing was carried out.

To test for nonrandom associations among aggregated phenotypes (antibiotic resistance profile, phage resistance profile, and plasmid profile), we used partial Mantel tests to test for correlations while correcting for phylogeny. These tests have been reported to have inflated false-positive rates under some conditions due to the permutation method used (58). Therefore, we used the phylogenetic permutation method according to the method of Harmon and Glor, which has been shown to overcome the type I error inflation seen with other methods for permuting the partial Mantel test (25), although this method was not assessed by Guillot and Rousset (58). We tested associations between individual pairs of resistance phenotypes across isolates, or between resistance phenotypes and plasmid replicons across isolates, while accounting for phylogenetic similarity, by assessing the nonparametric (Kendall) correlation between the phylogenetically independent contrasts (PICs) (26, 59) for each trait. We chose a nonparametric test due to the nonnormal distribution of some resistance phenotypes and Kendall’s tau in particular because it is less sensitive to individual points with large differences in ranks than alternative measures (60). We tested whether these pairwise associations between phylogenetically independent contrasts were on average stronger for antibiotic-antibiotic and phage-phage combinations than for antibiotic-phage combinations using the Kruskal-Wallis rank sum test. Finally, for principal-component analysis (R base package), we input resistance data (IC_90_ or ratio of growth with phage to that without phage) as correlation matrices to account for the different scales of the resistance measures for antibiotics and phages.

### Data availability.

Raw phenotypic data are available at Dryad (doi:10.5061/dryad.gj318) and genomic data are available at Enterobase (see Text S1).

10.1128/mBio.01341-17.10 TABLE S7  Mutations (from BW25113 reference) found in sequenced populations resistant to HK578 (Res1 to Res8) or their Δ*rfaF* ancestor. Download TABLE S7, DOCX file, 0.1 MB. Copyright © 2017 Allen et al. 2017 Allen et al. This content is distributed under the terms of the Creative Commons Attribution 4.0 International license.
