# Supplementary material for: Associations among Antibiotic and Phage Resistance Phenotypes in Natural and Clinical Escherichia coli Isolates
Source: mBio. 2017 Oct 31;8(5):e01341-17. doi: 10.1128/mBio.01341-17 (PMC5666156; doi:10.1128/mBio.01341-17)
Supplement: TABLE S1 [file mbo005173571st1.docx]

| **Name** | **Family** | **Receptor** | ***E. coli* host** | **Other notes** |
| --- | --- | --- | --- | --- |
| M13 | *Inoviridae* | F pilus | MG1655\|F+ | Filamentous |
| Q**β** | *Leviviridae* | F pilus | MG1655\|F+ | Unstable at 4^o^C; store at -80^o^C |
| U3 | *Microviridae* | LPS (Gal+) | MG1655 | Needs 1M NaCl for 4^o^C storage |
| BW-1 | *Myoviridae* | Unknown | MG1655 |  |
| RB69 | *Myoviridae* | OmpF/fhuA/waaG/WaaP | MG1655 |  |
| T4 | *Myoviridae* | OmpC or LPS | MG1655 |  |
| T6 | *Myoviridae* | Tsx | MG1655 |  |
| N4 | *Podoviridae* | NfrA | MG1655 |  |
| T7 | *Podoviridae* | LPS core | MG1655 |  |
| Ω8 | *Podoviridae* | LPS O8 antigen | ECOR 7 | To find host for culturing we searched for the O8 antigen in the ECOR collection (1). |
| HK578 | *Siphoviridae* | Unknown | MG1655 |  |
| λ_vir_ | *Siphoviridae* | LamB porin | MG1655 | Lytic variant cI26 |
| T5 | *Siphoviridae* | FhuA or LPS | MG1655 |  |
| PRD1 | *Tectiviridae* | IncW, P, or N plasmids | MG1655\|R388+ | Plasmid-dependent |

1. **Amor K**, **Heinrichs DE**, **Frirdich E**, **Ziebell K**, **Johnson RP**, **Whitfield C**. 2000. Distribution of core oligosaccharide types in lipopolysaccharides from *Escherichia coli*. Infection and Immunity **68**:1116–1124.
