# Supplementary material for: Associations among Antibiotic and Phage Resistance Phenotypes in Natural and Clinical Escherichia coli Isolates
Source: mBio. 2017 Oct 31;8(5):e01341-17. doi: 10.1128/mBio.01341-17 (PMC5666156; doi:10.1128/mBio.01341-17)
Supplement: TABLE S2 [file mbo005173571st2.docx]

| **Antibiotic** | **Class** | **Solvent** | **Breakpoint used (μg/ml)** | **Concentration range used (μg/ml)** |
| --- | --- | --- | --- | --- |
| Gentamicin | Aminoglycoside | dH_2_O | **2**-4 | 0.5-8 |
| Cefotaxime | Cephalosporin | dH_2_0 | **1**-2 | 0.25-4 |
| Chloramphenicol | Chloramphenicol | EtOH | **8** | 1-16 |
| Trimethoprim | DHFR inhibitor | DMSO | **2**-4 | 0.5-8 |
| Ciprofloxacin | Fluroquinalone | HCl 0.1M | 0.25-**0.5** | 0.125-2 |
| Tigecycline | Glycylcycline | DMSO | 1-**2** | 0.5-8 |
| Erythromycin | Macrolide | EtOH | **32-**64^a^ | 4-64 |
| Amoxicillin | Penicillin | dH_2_O | **8** | 2-32 |
| Polymyxin B | Polymyxin | dH_2_O | **2** (Colistin) | 0.25-4 |
| Rifampicin | Rifamycin | DMSO | **8**^a^ | 2-32 |
