# Supplementary figures and images for: Associations among Antibiotic and Phage Resistance Phenotypes in Natural and Clinical Escherichia coli Isolates
Source: mBio. 2017 Oct 31;8(5):e01341-17. doi: 10.1128/mBio.01341-17 (PMC5666156; doi:10.1128/mBio.01341-17)

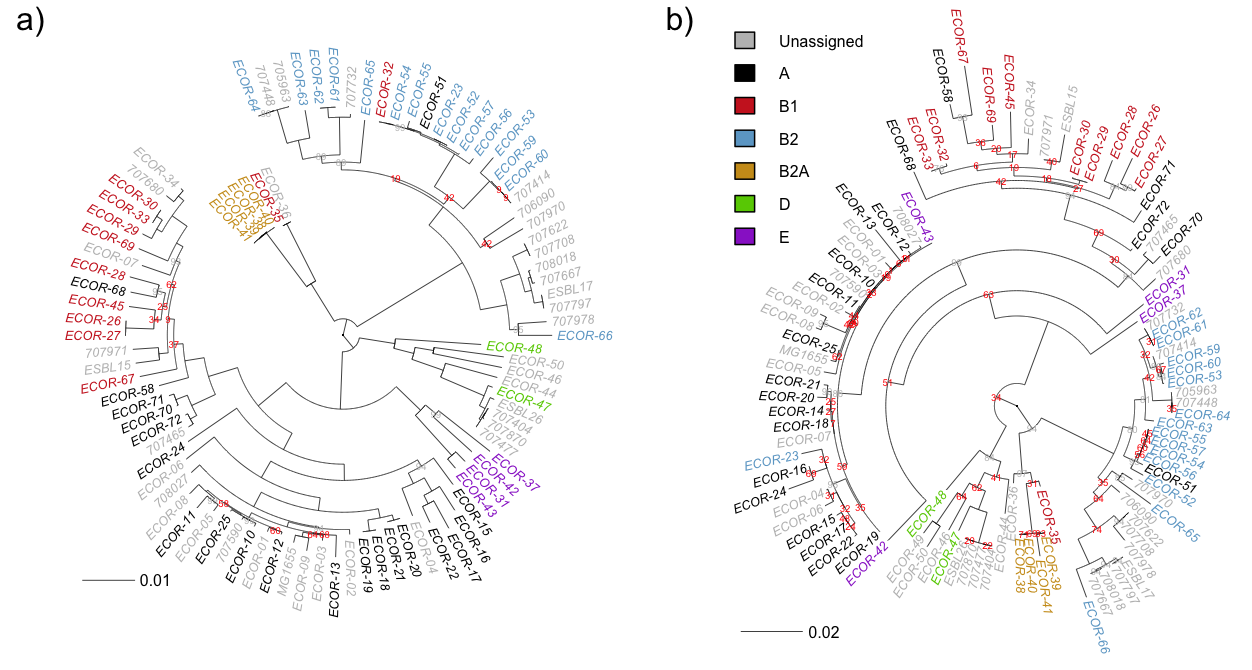

Supplement: FIG S1 [file mbo005173571sf1.tif]

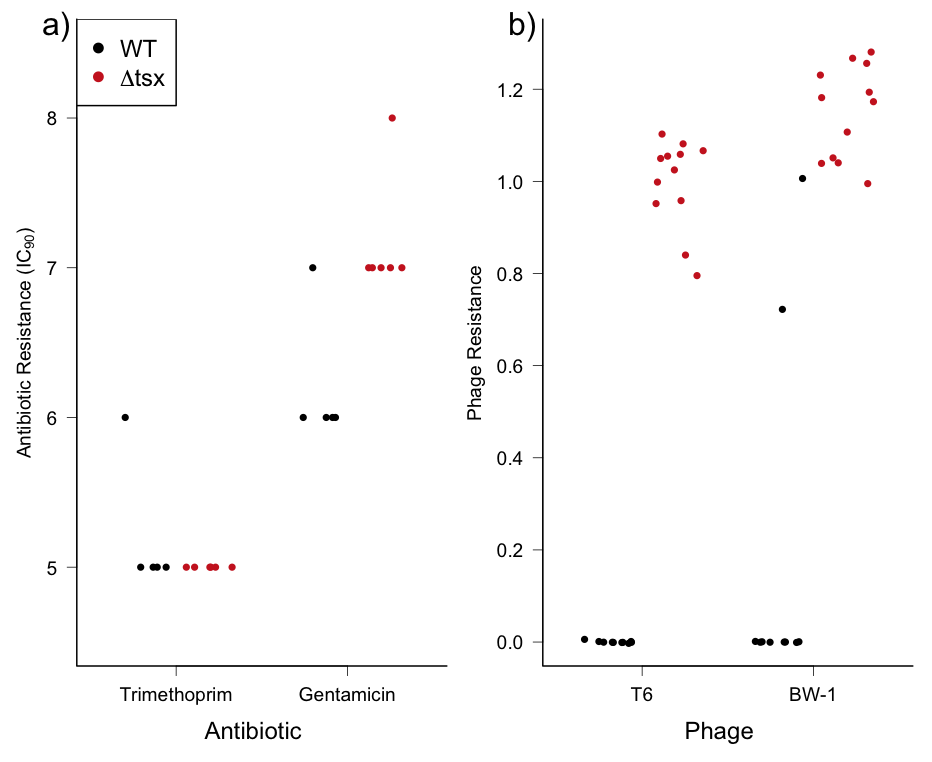

Supplement: FIG S2 [file mbo005173571sf2.tif]
