# Supplementary material for: Associations among Antibiotic and Phage Resistance Phenotypes in Natural and Clinical Escherichia coli Isolates
Source: mBio. 2017 Oct 31;8(5):e01341-17. doi: 10.1128/mBio.01341-17 (PMC5666156; doi:10.1128/mBio.01341-17)
Supplement: TEXT S1 [file mbo005173571s1.docx]

Text S1: Supplementary materials and methods

### Measuring susceptibility to phages and antibiotics

For each antibiotic×isolate combination, we estimated resistance from the growth inhibition observed across five concentrations of a two-fold broth-dilution series, measuring bacterial growth as optical density (OD_600_) using a Tecan Infinite M200 Pro plate spectrophotometer (Tecan; Männedorf, Switzerland). The midpoint of these ranges approximates the relevant sensitivity breakpoints taken from EUCAST and primary literature (Table S2 (1, 2)).

We measured resistance of every isolate to each phage and each antibiotic once in each of three blocks of assays. In each block of assays, we allocated each isolate a different, randomized microplate-well position. In a given block, we first grew 88 independent overnight cultures of each isolate in 1/10 LB (100μl per well in 88 separate microplates). From these 88 microplates, each containing one culture of every isolate, we inoculated 88 assay microplates, each containing LB with divalents (150μl per well) supplemented with bacteriophages (*n*=14 microplates, one for each phage species), antibiotics (*n*=5 concentrations×10 antibiotics=50 microplates) or unsupplemented (*n*=24 microplates). We inoculated the assay microplates in a randomised order, one from each of the 88 overnight microplates using a pin replicator to transfer 1μl of overnight culture per well to the assay microplates. We then incubated assay microplates for 20 hours without shaking, before lightly shaking them in the spectrophotometer and measuring optical density (OD_600_). Each measurement was corrected by subtracting the score for sterile media incubated in the same replicate of the experiment. Experiments using knockout strains from the Keio collection used the same methods, with similar steps taken to ensure randomisation and independence. Data in figure 4a were taken after 24 hours growth.

We found that a small fraction of control wells were false-inoculated (OD<0.05 despite no exposure to phages or antibiotics) and we excluded these from our estimation of control growth scores. Note that our criterion for IC_90_ determination also minimizes the influence of any false inoculation in that a concentration resulting in >90% inhibition is only assigned as the IC_90_ if inhibition is >90% at all of the higher concentrations as well. In phage experiments, failed inoculation of wells exposed to phages could potentially generate a false signal of strong inhibition by phages; we identified such cases by comparing the three independent replicates for each phage×isolate combination. In cases where some replicates attained positive growth (OD>0.05) in the presence of phages and others did not, we performed two additional independent replicates and accepted the three or four replicates that were in agreement regarding growth viability (OD > or < 0.05 in all) (3).

In further analyses, including testing for correlations among resistance phenotypes, we took resistance for each isolate×phage or isolate×antibiotic combination as the median of the three or four independent replicates.

## Plaquing for F-plasmid-dependent phages

Among isolates tested we did not find any that were susceptible to F-plasmid-dependent phages in liquid culture. To confirm this result, we tested whether M13 and Qβ phage could form plaques on LB overlay plates, each containing a different strain from the isolate collection. Consistent with our results in liquid culture, neither M13 or Qβ phage formed visible plaques on any of the 94 isolates, though as noted in the main text we did obtain plaques with an F+ version of K12 MG1655.

## Genome sequences of natural and clinical isolates

For the Basel isolates and for ECOR 72 (for which no assembly was available), we carried out whole-genome sequencing at the University Hospital Basel. DNA was extracted by using the EZ1 Advanced extraction robot (Qiagen), followed by DNA quality control measurements, library preparation (Nextera XT, Illumina). Library quality was assessed using Qubit 3.0 (ThermoFisher) and Tapestation (Agilent) assays. Libraries were sequenced on a MiSeq Illumina device using paired end mode with 2x300 nucleotide long reads and coverage >40-fold. We then uploaded raw reads to Enterobase for assembly so that the same assembly pipeline was used for all isolates. Assemblies can be downloaded easily from Enterobase (<http://enterobase.warwick.ac.uk/species/index/ecoli)> using the identifiers used in this paper as the name in the search strain function. However the assemblies for the ECOR strains are named as ECOR-## and our upload of ECOR 72 is listed as “ECOR-72” to match this convention.

## Phylogenetic reconstruction

We used the pipeline at Enterobase to obtain core genome multi-locus sequence typing (cgMLST) profiles for all isolates (4). 1424 loci from the Enterobase cgMLST scheme had nucleotide sequences for all isolates. We used a short R script to convert allele identities for these loci into named lists of allele sequences for each loci. We then used CLC Genomics (version 9.5.2 <https://www.qiagenbioinformatics.com/>) to align the allele lists separately, before concatenating the 1424 alignments into a single alignment 1,110,238bp long. From this alignment we generated an unrooted tree using RAxML (5) under default parameters run using the CIPRES (cyberinfrastructure for phylogenetic research) portal. Typical outgroup species such as *E. fergusonii* (6) lacked sequences for many of the 1424 loci used here. Therefore we manually set the root in our cgMLST tree to the same position as in the Pasteur MLST tree (see below)

(7).

We compared the results obtained with our cgMLST tree to those obtained with a common alternative method of MLST-tree construction, using the Pasteur MLST scheme (8, 9) with seven loci (*dinB*, *icdA*, *pabB*, *polB*, *putP*, *trpA*, *trpB*) that had nucleotide sequences for all the isolates in our library and the *E. fergusonii* outgroup. For this tree, we obtained allele sequences from the Pasteur database, filling gaps manually from sequence data. Again we aligned each locus separately and then concatenated the seven alignments. We then generated a rooted tree in RaxML using *E. fergusonii* as the outgroup, as has been done previously (6).

## Assessing sensitivity of correlations to rare sensitive isolates

Most phages only infected a subset of the isolates in our collection. Bacterial sensitivity (identified as a resistance score of <0.6) was rare (≤5 isolates) for one or both of the phages in three of the combinations of stressors where we observed a significant association across isolates. These were all correlations between two phages (correlations with antibiotic-resistance phenotypes involved phage T6, which was infectious against several isolates). We therefore tested the sensitivity of these correlations to exclusion of individual sensitive isolates, to identify cases where associations were driven by individual isolates. We did this by independently removing each sensitive isolate from the phenotypic data and from the phylogenetic tree. We then recalculated the phylogenetically independent contrasts and re-tested for a non-parametric (Kendall) correlation between the PICs of the two phenotypes. The *P*-values were then multiplied by the number of tests for the analysis of individual correlations (276), this is conservative but using sequential Bonferroni does not affect significance. The effects of removing individual isolates are shown in Table S3. Two correlations always became non-significant when a sensitive strain was removed and the third was relatively robust to removal of sensitive strains, though in all cases changes in the strength of the association (Kendall's Tau) were small.

## Plasmid replicon profiles

We used PlasmidFinder (10, 11) on the genome assemblies for each isolate (with an identity threshold of 75%). The MG1655 genome (downloaded from NCBI) returned no hits, which is unsurprising given that it is known to be plasmid-free (12). After grouping by incompatibility (sub) types, we scored each isolate for presence or absence of nine incompatibility groups, four F-plasmid incompatibility subgroups and plasmids related to either p0111 or pSL483 (15 plasmid types in total). This included all incompatibility types returned by PlasmidFinder, covering many important resistance plasmids like F plasmids and L/M plasmids (13). The ECOR collection has already been typed for plasmids using molecular methods by Williams et. al. (14). This data set was significantly similar to the data set we obtained with PlasmidFinder: there was a significant correlation (Kendall) between presence/absence of the plasmid in our analysis and the analysis of Williams et al. across the seven plasmid types found in both datasets (IncF; Tau = 0.711, p<0.001; IncFIA, Tau = 0.55, p<0.001; IncFIB, Tau = 0.744, p<0.001; IncFIC, Tau = 1, p<0.001; IncX, Tau = 0.264, p<0.05; IncY, Tau = 0.859, P<0.001; IncI, Tau = 0.641, p<0.01) .

To identify candidate plasmids carrying gentamicin and trimethoprim resistance, the isolates with the highest level of both trimethoprim and gentamicin resistance (ESBL17 and 707622) were run through ResFinder (15) to identify relevant resistance genes. In each isolate, one candidate gene was found for each resistance type, on different assembly contigs. For each isolate we ran BLAST (in CLC genomics) on each contig to return the 100 NCBI entries with the closest matches to the contig. We compared the two lists of 100 sequences for sequences that had hits to both. Barring one sequence (CP003683 corresponding to a *Klebsiella michiganensis* chromosomal sequence) all sequences with matches to both regions were annotated as plasmid sequences. We then used PlasmidFinder to determine whether the replicons found in these sequences were also found in our isolates. The short list of plasmids with matches to both resistance regions for the two isolates is given in Tables S6.

## Genomic analysis of Phage-resistant mutants of *E. coli* BW25113

For the eight HK578-resistant mutants and their ∆*rfaF* ancestor we produced barcoded libraries with the NEBNext Ultra II DNA library prep kit, with a target insert size of 500bp. These were run through a MiSeq sequencer with 2x300bp paired end sequencing (600 cycles) using the Illumina MiSeq reagent kit v3. This was done at the Genetic Diversity Centre, ETH Zürich.

We used breseq (16, 17) to analyse the sequencing data, mapping paired reads to the annotated BW25113 reference genome. Several polymorphisms were assigned by the software but other junction evidence (adjacent parts of reads mapping to regions that are not adjacent in the reference), could not be assigned. These were manually resolved by examining the read evidence. To ensure that junction evidence had been correctly resolved the “gdtools APPLY” function of breseq was used to add the identified mutations to the reference genome and then the sequenced reads were run against that new reference to check that no mutations were identified (i.e. the reads exactly map the modified reference), as is recommended in breseq documentation (16). Figure 4b shows alleles not detected in the ancestral clone.

## Supplementary references

1. **Wiegand I**, **Hilpert K**, **Hancock REW**. 2008. Agar and broth dilution methods to determine the minimal inhibitory concentration (MIC) of antimicrobial substances. Nat Protoc **3**:163–175.

2. **Stock I**, **Wiedemann B**. 1999. Natural antibiotic susceptibility of *Escherichia coli*, *Shigella*, *E. vulneris*, and *E. hermannii* strains. Diagn Microbiol Infect Dis **33**:187–199.

3. **Imamovic L**, **Sommer MOA**. 2013. Use of collateral sensitivity networks to design drug cycling protocols that avoid resistance development. Science Translational Medicine **5**:204ra132–204ra132.

4. **Maiden MCJ**, **van Rensburg MJJ**, **Bray JE**, **Earle SG**, **Ford SA**, **Jolley KA**, **McCarthy ND**. 2013. MLST revisited: the gene-by-gene approach to bacterial genomics. Nature Publishing Group **11**:728–736.

5. **Stamatakis A**. 2014. RAxML version 8: a tool for phylogenetic analysis and post-analysis of large phylogenies. Bioinformatics **30**:1312–1313.

6. **Clermont O**, **Olier M**, **Hoede C**, **Diancourt L**, **Brisse S**, **Keroudean M**, **Glodt J**, **Picard B**, **Oswald E**, **Denamur E**. 2011. Animal and human pathogenic *Escherichia coli* strains share common genetic backgrounds. Infect Genet Evol **11**:654–662.

7. **Wielgoss SEB**, **Didelot X**, **Chaudhuri RR**, **Liu X**, **Weedall GD**, **Velicer GJ**, **Vos M**. 2016. A barrier to homologous recombination between sympatric strains of the cooperative soil bacterium Myxococcus xanthus **10**:2468–2477.

8. **Jaureguy F**, **Landraud L**, **Passet V**, **Diancourt L**, **Frapy E**, **Guigon G**, **Carbonnelle E**, **Lortholary O**, **Clermont O**, **Denamur E**, **Picard B**, **Nassif X**, **Brisse S**. 2008. Phylogenetic and genomic diversity of human bacteremic *Escherichia coli* strains. BMC Genomics **9**.

9. **Skurnik D**, **Clermont O**, **Guillard T**, **Launay A**, **Danilchanka O**, **Pons S**, **Diancourt L**, **Lebreton F**, **Kadlec K**, **Roux D**, **Jiang D**, **Dion S**, **Aschard H**, **Denamur M**, **Cywes-Bentley C**, **Schwarz S**, **Tenaillon O**, **Andremont A**, **Picard B**, **Mekalanos J**, **Brisse S**, **Denamur E**. 2015. Emergence of Antimicrobial-Resistant Escherichia coli of Animal Origin Spreading in Humans. Molecular Biology and Evolution msv280–17.

10. **Carattoli A**, **Zankari E**, **García-Fernández A**, **Voldby Larsen M**, **Lund O**, **Villa L**, **Møller Aarestrup F**, **Hasman H**. 2014. In silico detection and typing of plasmids using PlasmidFinder and plasmid multilocus sequence typing. Antimicrobial Agents and Chemotherapy **58**:3895–3903.

11. **Wong VK**, **Baker S**, **Pickard DJ**, **Parkhill J**, **Page AJ**, **Feasey NA**, **Kingsley RA**, **Thomson NR**, **Keane JA**, **Weill F-X**, **Edwards DJ**, **Hawkey J**, **Harris SR**, **Mather AE**, **Cain AK**, **Hadfield J**, **Hart PJ**, **Thieu NTV**, **Klemm EJ**, **Glinos DA**, **Breiman RF**, **Watson CH**, **Kariuki S**, **Gordon MA**, **Heyderman RS**, **Okoro C**, **Jacobs J**, **Lunguya O**, **Edmunds WJ**, **Msefula C**, **Chabalgoity JA**, **Kama M**, **Jenkins K**, **Dutta S**, **Marks F**, **Campos J**, **Thompson C**, **Obaro S**, **MacLennan CA**, **Dolecek C**, **Keddy KH**, **Smith AM**, **Parry CM**, **Karkey A**, **Mulholland EK**, **Campbell JI**, **Dongol S**, **Basnyat B**, **Dufour M**, **Bandaranayake D**, **Naseri TT**, **Singh SP**, **Hatta M**, **Newton P**, **Onsare RS**, **Isaia L**, **Dance D**, **Davong V**, **Thwaites G**, **Wijedoru L**, **Crump JA**, **De Pinna E**, **Nair S**, **Nilles EJ**, **Thanh DP**, **Turner P**, **Soeng S**, **Valcanis M**, **Powling J**, **Dimovski K**, **Hogg G**, **Farrar J**, **Holt KE**, **Dougan G**. 2015. Phylogeographical analysis of the dominant multidrug-resistant H58 clade of *Salmonella* Typhi identifies inter- and intracontinental transmission events. Nature Genetics **47**:632–639.

12. **Blattner FR**, **Plunkett G**, **Bloch CA**, **Perna NT**, **Burland V**, **Riley M**, **Collado-Vides J**, **Glasner JD**, **Rode CK**, **Mayhew GF**, **Gregor J**, **Davis NW**, **Kirkpatrick HA**, **Goeden MA**, **Rose DJ**, **Mau B**, **Shao Y**. 1997. The complete genome sequence of *Escherichia coli* K-12. Science **277**:1453–1462.

13. **Carattoli A**. 2009. Resistance plasmid families in *Enterobacteriaceae*. Antimicrobial Agents and Chemotherapy **53**:2227–2238.

14. **Williams LE**, **Wireman J**, **Hilliard VC**, **Summers AO**. 2013. Large plasmids of *Escherichia coli* and *Salmonella* encode highly diverse arrays of accessory genes on common replicon families. Plasmid **69**:36–48.

15. **Zankari E**, **Hasman H**, **Cosentino S**, **Vestergaard M**, **Rasmussen S**, **Lund O**, **Aarestrup FM**, **Larsen MV**. 2012. Identification of acquired antimicrobial resistance genes. Journal of Antimicrobial Chemotherapy **67**:2640–2644.

16. **Deatherage DE**, **Barrick JE**. 2014. Identification of mutations in laboratory-evolved microbes from next-generation sequencing data using breseq. Methods Mol Biol **1151**:165–188.

17. **Barrick JE**, **Colburn G**, **Deatherage DE**, **Traverse CC**, **Strand MD**, **Borges JJ**, **Knoester DB**, **Reba A**, **Meyer AG**. 2014. Identifying structural variation in haploid microbial genomes from short-read resequencing data using breseq. BMC Genomics **15**.
