# Supplementary material for: Associations among Antibiotic and Phage Resistance Phenotypes in Natural and Clinical Escherichia coli Isolates
Source: mBio. 2017 Oct 31;8(5):e01341-17. doi: 10.1128/mBio.01341-17 (PMC5666156; doi:10.1128/mBio.01341-17)
Supplement: TABLE S3 [file mbo005173571st3.docx]

| Association | Excluded  Strain | Kendall’s  tau | *P*-value | Significance  (Corrected, n=276) |
| --- | --- | --- | --- | --- |
| HK578-U3 | *None* | 0.264 | 1.77E-04 | * |
|  | MG1655 | 0.233 | 9.95E-04 |  |
|  | ECOR13 | 0.251 | 3.98E-04 |  |
|  | ECOR4 | 0.230 | 1.18E-03 |  |
|  | ECOR55 | 0.262 | 2.19E-04 |  |
|  | ECOR11 | 0.234 | 9.49E-04 |  |
| PRD1-N4 | *None* | 0.273 | 1.03E-04 | * |
|  | ECOR9 | 0.256 | 3.08E-04 |  |
|  | ECOR16 | 0.288 | 4.89E-05 | * |
|  | ECOR64 | 0.248 | 4.64E-04 |  |
|  | ECOR28 | 0.295 | 3.15E-05 | * |
|  | MG1655 | 0.272 | 1.24E-04 | * |
|  | 707404 | 0.287 | 5.18E-05 | * |
| T5-T7 | *None* | 0.287 | 4.72E-05 | * |
|  | MG1655 | 0.216 | 2.30E-03 |  |
|  | ECOR4 | 0.214 | 2.57E-03 |  |
|  | ECOR16 | 0.280 | 7.72E-05 | * |
