# Supplementary material for: Associations among Antibiotic and Phage Resistance Phenotypes in Natural and Clinical Escherichia coli Isolates
Source: mBio. 2017 Oct 31;8(5):e01341-17. doi: 10.1128/mBio.01341-17 (PMC5666156; doi:10.1128/mBio.01341-17)
Supplement: TABLE S4 [file mbo005173571st4.docx]

| **Resistance phenotype** | **Correlation** | **P(r=0)** | **P.holm(r=0)** |
| --- | --- | --- | --- |
| Cefotaxime | 0.229 | 0.000 | 0.000 |
| Ciprofloxacin | 0.161 | 0.000 | 0.000 |
| Amoxicillin | 0.173 | 0.005 | 0.040 |
| Trimethoprim | 0.101 | 0.032 | 0.224 |
| Gentamicin | 0.017 | 0.355 | 1.000 |
| Chloramphenicol | 0.014 | 0.391 | 1.000 |
| Rifampicin | 0.005 | 0.433 | 1.000 |
| Erythromycin | -0.002 | 0.493 | 1.000 |
| Polymyxin B | -0.021 | 0.605 | 1.000 |
| Tigecycline | -0.018 | 0.669 | 1.000 |
