# Supplementary material for: Associations among Antibiotic and Phage Resistance Phenotypes in Natural and Clinical Escherichia coli Isolates
Source: mBio. 2017 Oct 31;8(5):e01341-17. doi: 10.1128/mBio.01341-17 (PMC5666156; doi:10.1128/mBio.01341-17)
Supplement: TABLE S5 [file mbo005173571st5.docx]

| **Antibiotic** | **Amoxicillin** | | **Cefotaxime** | | **Ciprofloxacin** | |
| --- | --- | --- | --- | --- | --- | --- |
| **Plasmid replicon** | **tau** | **P.holm (τ=0)** | **tau** | **P.holm (τ=0)** | **tau** | **P.holm (τ=0)** |
| Col | 0.204 | 0.078 | 0.176 | 0.338 | 0.070 | 1.000 |
| IncB/O/K/Z | -0.048 | 1.000 | 0.152 | 0.874 | 0.133 | 1.000 |
| IncFIA | -0.045 | 1.000 | 0.096 | 1.000 | 0.266 | 0.044 |
| IncFIB | 0.241 | 0.013 | 0.142 | 0.811 | 0.163 | 0.612 |
| IncFIC | 0.062 | 1.000 | 0.244 | 0.095 | 0.455 | 2.074x10^-5^ |
| IncFII | 0.131 | 0.936 | -0.001 | 1.000 | -0.091 | 1.000 |
| IncHI1B | -0.024 | 1.000 | 0.122 | 1.000 | -0.109 | 1.000 |
| IncI | 0.111 | 1.000 | 0.230 | 0.095 | 0.054 | 1.000 |
| IncL/M | 0.111 | 1.000 | -0.001 | 1.000 | -0.135 | 1.000 |
| IncN | 0.054 | 1.000 | 0.039 | 1.000 | -0.090 | 1.000 |
| IncQ | 0.109 | 1.000 | 0.007 | 1.000 | 0.008 | 1.000 |
| IncX | -0.103 | 1.000 | 0.053 | 1.000 | 0.101 | 1.000 |
| IncY | 0.012 | 1.000 | 0.027 | 1.000 | -0.068 | 1.000 |
| p0111 | 0.107 | 1.000 | 0.358 | 0.001 | 0.496 | 1.870x10^-6^ |
| pSL483 | 0.034 | 1.000 | 0.124 | 1.000 | -0.058 | 1.000 |
