# Supplementary material for: Associations among Antibiotic and Phage Resistance Phenotypes in Natural and Clinical Escherichia coli Isolates
Source: mBio. 2017 Oct 31;8(5):e01341-17. doi: 10.1128/mBio.01341-17 (PMC5666156; doi:10.1128/mBio.01341-17)
Supplement: TABLE S6 [file mbo005173571st6.docx]

| **Isolate** | **Accession** | **Trimethoprim resistance region (*dfrA1*)** | | | **Gentamicin resistance region** | | | | **Plasmid replicon profile** | | | | | | | **Plasmid replicons also present in isolate** |
| --- | --- | --- | --- | --- | --- | --- | --- | --- | --- | --- | --- | --- | --- | --- | --- | --- |
|  |  | **Blast Score** | **HSP length** | **Identity (%)** | **Resistance**  **gene** | **Blast Score** | **HSP length** | **Identity (%)** | **Col** | **IncFIA** | **IncFIB** | **IncFIC** | **IncFII** | **IncQ** | **IncR** |  |
| ESBL17 | CP010149 | 21232 | 10679 | 99.99 | *aac(3)-IIa* | 2664 | 1460 | 96.51 | 0 | 0 | 1 | 1 | 0 | 0 | 1 | 2/3 |
| ESBL17 | CP010174 | 20982 | 10549 | 100.00 | *aac(3)-IIa* | 5458 | 2729 | 100.00 | 0 | 0 | 0 | 0 | 1 | 0 | 1 | 1/2 |
| ESBL17 | CP012197 | 21234 | 10678 | 99.99 | *aac(3)-IIa* | 5708 | 2854 | 100.00 | 1 | 1 | 1 | 0 | 1 | 0 | 0 | 4/4 |
| ESBL17 | CP018365 | 420 | 662 | 73.95 | *aac(3)-IIa* | 3618 | 1809 | 100.00 | 0 | 0 | 1 | 0 | 1 | 0 | 1 | 2/3 |
| ESBL17 | FQ482074 | 20866 | 10500 | 99.98 | *aac(3)-IIa* | 5070 | 2535 | 100.00 | 0 | 0 | 1 | 0 | 1 | 1 | 0 | 2/3 |
| ESBL17 | LN850163 | 20980 | 10553 | 99.98 | *aac(3)-IIa* | 2664 | 1460 | 96.51 | 0 | 1 | 1 | 0 | 1 | 0 | 0 | 3/3 |
| 707622 | CP003683 | 20058 | 10096 | 99.96 | *aac(3)-IId* | 4296 | 2148 | 100.00 | 0 | 0 | 0 | 0 | 0 | 1 | 0 | 0/1 |
| 707622 | CP010138 | 20068 | 10097 | 99.98 | *aac(3)-IId* | 4038 | 2024 | 99.90 | 0 | 0 | 0 | 0 | 1 | 0 | 0 | 1/1 |
| 707622 | CP010149 | 20060 | 10098 | 99.97 | *aac(3)-IId* | 4278 | 2149 | 99.86 | 0 | 0 | 1 | 1 | 0 | 0 | 1 | 1/3 |
| 707622 | CP014498 | 20324 | 10220 | 100.00 | *aac(3)-IId* | 4292 | 2151 | 99.91 | 1 | 1 | 1 | 0 | 1 | 0 | 0 | 3/4 |
| 707622 | CP018982 | 20324 | 10220 | 100.00 | *aac(3)-IId* | 3868 | 1937 | 99.95 | 0 | 1 | 1 | 0 | 1 | 0 | 0 | 2/3 |
| 707622 | CP019009 | 20324 | 10220 | 100.00 | *aac(3)-IId* | 4292 | 2151 | 99.91 | 0 | 1 | 1 | 0 | 1 | 0 | 0 | 2/3 |
| 707622 | FJ876827 | 20324 | 10220 | 100.00 | *aac(3)-IId* | 4534 | 2275 | 99.87 | 1 | 1 | 1 | 0 | 1 | 0 | 0 | 3/4 |
| 707622 | LN850163 | 19812 | 9972 | 99.97 | *aac(3)-IId* | 2024 | 1012 | 100.00 | 0 | 1 | 1 | 0 | 1 | 0 | 0 | 2/3 |
