# Supplementary material for: Associations among Antibiotic and Phage Resistance Phenotypes in Natural and Clinical Escherichia coli Isolates
Source: mBio. 2017 Oct 31;8(5):e01341-17. doi: 10.1128/mBio.01341-17 (PMC5666156; doi:10.1128/mBio.01341-17)
Supplement: TABLE S7 [file mbo005173571st7.docx]

| **Position** | **Mutation** | **Type** | **Presence of mutation** | | | | | | | | | **Annotation** | **Gene** |
| --- | --- | --- | --- | --- | --- | --- | --- | --- | --- | --- | --- | --- | --- |
|  |  |  | **Res1** | **Res2** | **Res3** | **Res4** | **Res5** | **Res6** | **Res7** | **Res8** | **𝚫rfaF ancestor** |  |  |
| 270,861 | Δ28,438 bp | DEL |  |  |  |  |  | 1 |  |  |  | IS5‑mediated | *mmuP*–[*yagU*] |
| 360,104 | 183 bp x 3 | AMP | 1 | 1 | 1 | 1 | 1 | 1 | 1 | 1 | 1 | amplification | *lacZ*← / ← *lacI* |
| 454,246 | IS186 (–) +6 bp :: Δ1 bp | MOB |  | 1 | 1 |  |  | 1 |  |  |  | intergenic (+90/‑93) | *clpX*→ / → *lon* |
| 454,246 | IS186 (+) +6 bp :: Δ1 bp | MOB | 1 |  |  | 1 | 1 |  | 1 | 1 |  | intergenic (+90/‑93) | *clpX*→ / → *lon* |
| 634,031 | A→T | SNP |  |  | 1 |  |  |  |  |  |  | intergenic (‑2/‑370) | *dsbG*← / → *ahpC* |
| 1,193,961 | (AATGATGA)6→7 | INS |  | 1 |  |  |  |  |  |  |  | intergenic (‑268/‑190) | *ymfE*← / → *lit* |
| 1,203,262 | 1,797 bp inversion | INV | 1 | 1 | 1 |  |  | 1 |  |  | Polymorphic | pseudogene (18/501 nt) | *stfP*↓*stfE* |
| 1,205,059 | 16 bp→CP009273:1203261‑1203246 | CON | 1 | 1 | 1 |  |  | 1 |  |  | Polymorphic | pseudogene (2‑17/501 nt) | *stfE*← |
| 3,788,202 | Δ1,235 bp | DEL | 1 | 1 | 1 | 1 | 1 | 1 | 1 | 1 | 1 |  | [*hldD*]–[*waaC*] |
| 4,532,125 | IS5 (+) +4 bp | MOB |  | 1 | 1 |  |  |  | 1 |  | Polymorphic | coding (272‑275/597 nt) | *fimE*→ |
| 4,532,508 | 296 bp inversion | INV |  |  | 1 |  |  |  | 1 |  | Polymorphic | intergenic (+353/‑129) | *fimE*/*fimA*↓*fimE*/*fimA* |
| 4,532,542 | (T)6→5 | SND | 1 |  |  | 1 | 1 | 1 |  | 1 |  | intergenic (+92/‑390) | *fimE*→ / → *fimA* |
| 4,532,804 | 9 bp→CP009273:4532507‑4532499 | CON |  |  | 1 |  |  |  | 1 |  | Polymorphic | intergenic (+354/‑120) | *fimE*→ / → *fimA* |
